# Effects of training and using an audio-tactile sensory substitution device on speech-in-noise understanding

**DOI:** 10.1038/s41598-022-06855-8

**Published:** 2022-02-25

**Authors:** K. Cieśla, T. Wolak, A. Lorens, M. Mentzel, H. Skarżyński, A. Amedi

**Affiliations:** 1 The Baruch Ivcher Institute for Brain, Cognition & Technology, The Baruch Ivcher School of Psychology and the Ruth and Meir Rosental Brain Imaging Center, Reichman University, Herzliya, Israel; 2 World Hearing Centre, Institute of Physiology and Pathology of Hearing, Warsaw, Poland

**Keywords:** Neuroscience, Health care

## Abstract

Understanding speech in background noise is challenging. Wearing face-masks, imposed by the COVID19-pandemics, makes it even harder. We developed a multi-sensory setup, including a sensory substitution device (SSD) that can deliver speech simultaneously through audition and as vibrations on the fingertips. The vibrations correspond to low frequencies extracted from the speech input. We trained two groups of non-native English speakers in understanding distorted speech in noise. After a short session (30–45 min) of repeating sentences, with or without concurrent matching vibrations, we showed comparable mean group improvement of 14–16 dB in Speech Reception Threshold (SRT) in two test conditions, i.e., when the participants were asked to repeat sentences only from hearing and also when matching vibrations on fingertips were present. This is a very strong effect, if one considers that a 10 dB difference corresponds to doubling of the perceived loudness. The number of sentence repetitions needed for both types of training to complete the task was comparable. Meanwhile, the mean group SNR for the audio-tactile training (14.7 ± 8.7) was significantly lower (harder) than for the auditory training (23.9 ± 11.8), which indicates a potential facilitating effect of the added vibrations. In addition, both before and after training most of the participants (70–80%) showed better performance (by mean 4–6 dB) in speech-in-noise understanding when the audio sentences were accompanied with matching vibrations. This is the same magnitude of multisensory benefit that we reported, with no training at all, in our previous study using the same experimental procedures. After training, performance in this test condition was also best in both groups (SRT ~ 2 dB). The least significant effect of both training types was found in the third test condition, i.e. when participants were repeating sentences accompanied with non-matching tactile vibrations and the performance in this condition was also poorest after training. The results indicate that both types of training may remove some level of difficulty in sound perception, which might enable a more proper use of speech inputs delivered via vibrotactile stimulation. We discuss the implications of these novel findings with respect to basic science. In particular, we show that even in adulthood, i.e. long after the classical “critical periods” of development have passed, a new pairing between a certain computation (here, speech processing) and an atypical sensory modality (here, touch) can be established and trained, and that this process can be rapid and intuitive. We further present possible applications of our training program and the SSD for auditory rehabilitation in patients with hearing (and sight) deficits, as well as healthy individuals in suboptimal acoustic situations.

## Introduction

Access to speech reading and efficient integration of visual information with auditory input is essential for enhanced understanding of speech to people with hearing loss, who naturally receive degraded auditory cues from the environment. Meanwhile, the COVID19 pandemic imposed on us the obligation of social distancing and wearing masks covering the mouth. Both these restrictions reduce transmission of sounds and prevent access to visual cues from speech/lip-reading, thereby further aggravating these difficulties^1,2^. Healthy individuals also use lip-reading in daily face-to-face communication, and especially benefit from it when the acoustic context is ambiguous, such as when exposed to rapid speech, speech in a non-native language, background noise and/or multiple speakers talking simultaneously^3–5^. With visual cues present, including both lip-reading and watching gestures, understanding speech against noise has been consistently found to improve, in both healthy individuals and in patients with hearing loss^1,6–10^.

In today’s modern society challenging acoustic conditions occur increasingly more often, including exposure to multiple concurrent auditory streams and almost constant exposure to noise. At the same time, the prevalence of hearing loss is growing (WHO 2020), condition which if left untreated, can accelerate cognitive decline, social isolation and depression^11–14^. In addition, many users of modern hearing aids and cochlear implants complain that their devices fail to effectively compensate for their hearing loss when they are exposed to ambiguous acoustic situations^15–17^.

All this indicates the importance of developing novel training methods and devices that can be employed to improve communication. Especially solutions combining multisensory inputs are appealing, as increasingly more experimental studies^18–20^ and conceptual works point to the superiority of multisensory over unisensory environments for learning and sensory recovery^21–25^. Multisensory speech training regimes that complement audition with vision have been found successful^10,25–27^, including in rehabilitation of patients with hearing aids (HA) and/or cochlear implants (CI), by adding speech reading, gestures or sign language cues^1,6,28^.

Several recent works showed benefits of providing tactile (instead of visual) stimulation corresponding to low-frequencies of speech, to improve comprehension of distorted auditory speech in noise, including our own findings^29–31^. The idea of adding low-frequency *tactile* information to improve speech perception was first inspired by studies showing the benefit of complementing degraded speech signals with low-frequency *auditory* information (e.g. when using both a hearing aid and a cochlear implant), which carries pitch information and thus helps segregate auditory streams, including discriminating between speakers^29,32,33^.

Applying a combination of auditory and tactile stimulation in assistive communication devices is an interesting and novel approach, that can prove useful especially if access to visual cues during communication is limited or absent (which apart from the discussed cases is also the every-day reality of the visually impaired). Audition and touch share some key features, such that both use mechanoreceptors to encode vibration in a common range of low frequencies (approximately 50 Hz to 700 Hz). Therefore, vibrotactile and auditory information can be naturally perceived as an interleaved signal, which is then also processed in partially shared brain regions, in both hearing participants as well as in congenitally deaf^34–40^. Auditory and vibrotactile inputs are also often experienced together, such as when a mobile phone is ringing and buzzing or when driving a car.

Given these similarities between senses, we developed an in-house audio-to-touch (assistive) Sensory Substitution Device (SSD). SSDs convey information typically delivered by one sensory modality (e.g., vision) through a different sensory modality (audition, touch) using specific translation algorithms that can be learned by the user^41–44^. A classic example is a chair developed by Prof Bach-Y-Rita in 1969, that delivered a visual image to the back of its blind users, through patterns of vibration^43^.

In our first work with the audio-to-touch SSD we showed immediate and robust improvement of 6 dB on average in healthy subjects in speech-in-noise understanding (Speech Reception Threshold, SRT), when auditory speech was complemented with low-frequency tactile vibrations delivered on fingertips^31^. Importantly, in our previous experiment the improvement occurred without applying any training. This immediate effect was in contrast to a number of other works using other SSDs, including devices translating vision to sound or vision to touch, which required hours of training and/or prolonged use to yield benefits, probably due to the complexity of the applied algorithms^43,45–49^.

In the current study we sought to investigate whether we can replicate our previous findings of showing improved understanding of distorted speech in noise when the signal is accompanied with concurrent vibrations on fingertips. We also applied two types of training, unisensory auditory training and multisensory audio-tactile training, to see whether performance can improve even further. We also wanted to determine how the training will impact understanding of completely novel sentences in three test conditions, with or without concurrent matching (corresponding to the audio sentence) or non-matching vibrations delivered on fingertips with our SSD. To our knowledge, this is the first study exploring speech-to-touch sensory substitution that also applied a control training session and a control multisensory speech test condition^29,30^.

We chose to study non-native English speakers with good levels of English in order to take advantage of the inverse effectiveness principle of multisensory integration, according to which multisensory enhancement (here, adding matching tactile input to a degraded auditory speech signal) is strongest when the ratio is lowest between senses^50–52^. In the current study we made the auditory task challenging by introducing distortions to resemble hearing through a cochlear implant (noise-vocoding^30,53^), presenting the sentences with background speech noise and in addition using a non-native language of the participants. Several studies have shown that listeners have more difficulty perceiving non-native speech in noise than the native speakers, despite similar performance in a quiet environment^10,54–56^, and at the same time might more readily benefit from multisensory speech cues^10,27,56–58^.

The objectives of this study are important for both practical reasons and for enhancing basic science understanding of multisensory processing and cross-modal integration. The audio-tactile multisensory context of speech using audio-tactile sensory substitution is especially intriguing, as it is completely novel to the study participants and only experienced during participation in the experiment. This contrasts with audio-visual speech, i.e. listening to speech and lip reading/observing gestures at the same time, which is the natural multisensory input to which we are all exposed from early development and throughout the lifetime (see review in^9^). If our SSD solution and training proves effective in the multisensory context, this will indicate that even in adulthood a new pairing between a certain computation (here, speech processing) and an atypical sensory modality (here, touch) can be built. This effect can expand the classical notion of closed “critical periods” for sensory development in childhood. We hypothesized that with speech being the most relevant biological signal for human and due to the similarities between the two employed senses to convey it, we will see rapid and profound perceptual effects of training. We furthermore hypothesized that multisensory audio-tactile training will be superior to unisensory training, through cross-modal enhancement. Potential applications of our set-up include auditory speech rehabilitation in the hearing impaired (but also training audio-tactile integration in late visual impairments^59^) and assisting healthy individuals in suboptimal acoustic contexts, including when learning a foreign language, listening to music or talking on the phone.

## Material and methods

### Material

Forty (N = 40) participants took part in the study (19 male, 21 female, age: M = 26.67, SD = 3.48 years), mostly University students or their friends. All participants completed an in-house questionnaire asking basic questions about their Hebrew and English language background. They were all native Hebrew speakers (born in Israel and learned Hebrew as their first language), right-handed, and reported no history of neurological or neurodevelopmental impairments. For all participants English was their first foreign language and they knew it well (their total mean self-rated English proficiency was 4.26 ± 0.35 on the scale from 1 to 5, see Table 1) and started to learn English at a relatively young age (to speak English at a mean age of 6.85 ± 2.53 years; 95% started learning before age 9 and 57% before age 7; to write in English at the mean age of 7.7 ± 1.81 years, with 92.5% before age 9). They communicated in English approximately 20% of a regular day (mean group results, Table 1). All participants confirmed that they had age-normal hearing (pure tone audiometry thresholds from their personal medical records < 20 dB for 0.125–8 kHz). In order to examine the effects of applying two types of training of speech understanding, unisensory and multisensory, the participants were divided into two groups of 20 participants. The groups were of similar age and gender composition, English language proficiency and age when they started learning English (see Table 1 for results of F-tests ad Chi^2^ tests). Group 1 (N = 20, 10 male/10 female; mean age = 27.8 ± 3.53) participated in a multisensory (audio-tactile) training session, in which they were repeating sentences presented to them both through headphones and as a corresponding matching vibrotactile input on fingertips (see “Methods” for the details of the procedure). Group 2 had training with the sentences presented solely through audition (N = 20, 11 male/9 female; mean age = 25.55 ± 3.11).

**Table 1:** Group composition and the English language background.

	Group 1 & group 2	Group 1: trained with audio-tactile input	Group 2: trained with audio only input	Between-group comparisons
N	40	20	20	N.A
Age	M = 26.67, SD = 3.48	M = 27.8, SD = 3.53	M = 25.55, SD = 3.11	F(1,38) = 0.01, p = 0.897
Female:male	21:19	10:10	9:11	χ^2^ = 0.05, p = 0.752
Age of initial English language acquisition “At what age did you start learning English? “	M = 6.85, SD = 2.53	M = 6.95, SD = 2.78	M = 6.75, SD = 2.33	F(1,38) = 0.26, p = 0.612
Years of using/being exposed to the English language	M = 19.82, SD = 4.3	M = 20.84, SD = 4.1	M = 18.8, SD = 4.34	F(1,38) = 0.01, p = 0.911
Self-rated English proficiency (scale 1–5) “How would You rate your English abilities in …?”	(a) Reading: M = 4.43, SD = 0.59 (b) Writing: M = 4.03, SD = 0.66 (c) Speaking: M = 4.35, SD = 0.66 (d) Understanding from hearing: M = 4.45, SD = 0.67	(a) Reading: M = 4.45, SD = 0.68 (b) Writing: M = 3.9, SD = 0.85 (c) Speaking: M = 4.35, SD = 0.74 (d) Understanding from hearing: M = 4.45, SD = 0.68	(a) Reading: M = 4.4, SD = 0.5 (b) Writing: M = 4.15, SD = 0.36 (c) Speaking: M = 4.35, SD = 0.58 (d) Understanding from hearing: M = 4.45, SD = 0.86	(a) Reading: F(1,38) = 3.26, p = 0.079 (b) Writing: F(1,38) = 8.21, p = 0.07 (c) Speaking: F(1,38) = 1.98, p = 0.16 (d) Understanding from hearing: F(1,38) = 0.00, p = 1.000
Every-day communication in English “When communicating with others, what percentage of the time, during a typical day, do you communicate in English?	M = 21.78, SD = 21.61	M = 24.4, SD = 22.5	M = 19.15, SD = 20.9	F(1,38) = 0.58, p = 0.45

### Method

#### The experimental set-up and the speech-to-touch SSD

**Figure 1:**
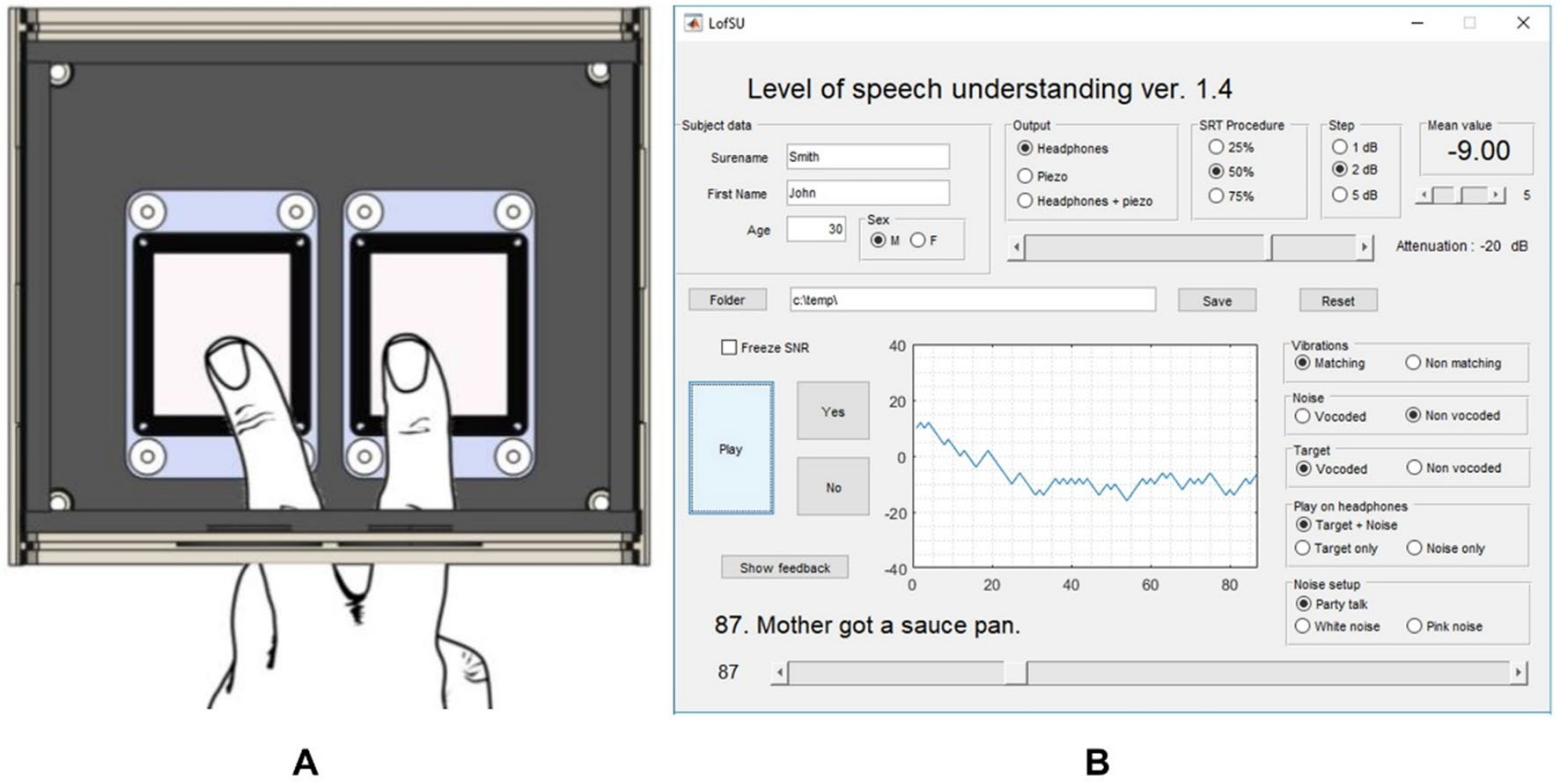
(**A**) The vibrating interface of the SSD and (**B**) the MatLab GUI.

To run the study, we developed a dedicated MatLab application (version R016a, The MathWorks Inc., Natick, MA, USA) with a user-friendly GUI (see Fig. 1B) that can be used to both establish SRT (Speech Reception Threshold, SRT, i.e., signal-to-noise ratio for 50% understanding) for a tested individual, as well as generate speech stimuli at certain signal-to-noise ratios for the training session, with or without concurrent vibrations delivered on fingertips. Our in-house audio-to-touch Sensory Substitution Device (SSD) (designed in collaboration with the World Hearing Centre in Warsaw, Poland and Neurodevice company [http://www.neurodevice.pl/en]) was used to deliver the speech signal as tactile vibration. The main part of the SSD is a vibrating interface with two piezoelectric plates, to simultaneously provide vibrotactile inputs on the index and middle finger of the dominant hand (Fig. 1A). The interface is connected to a controller powered from a socket of 230 V and vibration is delivered from a PC. The interface’s enclosure is isolated with foam to ensure some soundproofing and dampening. For auditory stimulation we used noise-cancelling headphones (BOSE QC35 IIA). Both the SSD and the headphones were connected to the PC through a 5.1 soundcard (Creative Labs, SB1095).

#### The speech stimuli

As speech stimuli we used recordings of the Hearing in Noise Test (HINT) sentences^60^ originally organized in 28 equivalent lists of 10 sentences that have been normalized for naturalness, difficulty, and intelligibility [in our database recordings of two sentences were missing, i.e. we had 278]. All sentences were of similar length (2.5–2.8 s) and conveyed a simple semantic content, such as e.g. “The boy fell from the window” or “It’s getting cold in here”. The energy of each sentence was normalized with a standard RMS procedure, peaks of energy were leveled to − 6 dB and the sounds were stored as 16-bit 44.1 kHz digital waveforms. Next, the sentences were noise-vocoded to resemble stimulation through a cochlear implant (CI) using an in-house algorithm developed at the Institute of Physiology and Pathology of Hearing^53^. The process of vocoding mainly involved band-pass filtering to 8 channels and modulation of the signal with a narrowband noise (the details of the applied algorithm were described in our previous work^31^). For all the testing conditions and during the Training session, the sentences were presented against background speech noise (International Female Fluctuating Noise, IFFN; https://www.ehima.com/).

#### The experiment timeline

**Figure 2:**
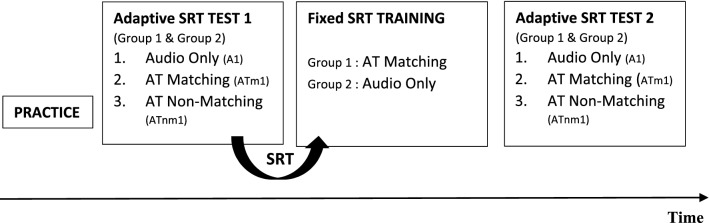
The timeline of the experiment. *AT* Audio-Tactile, *SRT* an individually established Speech Reception Threshold that is then used throughout the Training session.

After a short practice session, all participants took part in three tests of speech-in-noise comprehension to establish their individual SRTs, once before and once after a short training session, as depicted in Fig. 2.

##### Practice

All participants started with a brief practice session of listening to 2–4 different noise-vocoded and non-vocoded sentences from the English HINT sentence database^60^, with and without accompanying background noise and/or vibrations on fingertips, only to familiarize themselves with the study setting and the task.

##### Speech reception threshold tests

In the actual tests, as well as in the training session, the task of the participants was to repeat vocoded sentences in speech background noise. There were 3 test conditions, both before and after training, all with sentences presented in the headphones: (a) with no concurrent vibration delivered on fingertips (Audio Only, A), (b) together with low frequencies including the fundamental frequency (f0) extracted from the heard sentence, i.e. matching f0, delivered as vibration on fingertips (hereafter Audio-Tactile Matching, ATm), (c) together with low frequencies, including f0 not corresponding, i.e. non-matching, to the heard sentence, delivered as vibration on fingertips (hereafter, Audio-Tactile non-Matching, ATnm). Both study groups participated in all test conditions. In both groups, the A condition always came first, followed by the ATm condition in 10 out of 20 participants (A, ATm, ATnm), and by the ATnm condition in the remaining 10 participants (A, ATnm, ATm). As described in our previous work^31^, f0 had been earlier extracted from each original sentence derived from the English HINT database, using the STRAIGHT algorithm that was further improved. For the audio-tactile matching test condition the vibrotactile input represented low frequencies including f0 of the sentence presented in the headphones. For the audio-tactile non-matching test condition, the vibrotactile input represented low frequencies including f0 of another sentence randomly selected by a MATLAB algorithm from the HINT database. For each test we always used 20 different sentences (2 lists) that were presented in a fixed order of their appearance on the list. The difficulty level was adapted based on individual performance (2 dB up–2 dB down) during task performance. The outcome measure of each speech test was Speech Reception Threshold (SRT) of the target sentences against background noise, calculated as a mean of 5 last reversals. The applied adaptive procedure is typical for clinical settings when assessing speech understanding in hearing aid and/or cochlear implant users^61^. After the training session participants performed the second round of 3 speech test conditions in the same order as the first set of tests (A, ATm, ATnm or A, ATnm, ATm).

##### Training

After the initial series of 3 tests of speech understanding each person participated in a short training session. The training consisted of repeating 148 vocoded sentences in background speech noise. In Group 1 each sentence presented through the headphones during training was accompanied with matching low-frequency vibrotactile inputs delivered on fingertips of two fingers via our SSD. Group 2 participated in a training session with sentences presented through audition only. The SNR during training corresponded to the SRT value of a given person established before training for a given test condition and was kept constant during the whole training session. In Group 1 it was the SRT value established in the pre-training test condition involving auditory and matching tactile input (ATm). In Group 2, the SRT for the training corresponded to the SRT obtained by the person in the pre-training Audio only (A) test condition. During the training session (30–50 min in duration), each sentence was presented up to 3 times in a row and if the person was unable to repeat it correctly, the sentence was presented as text on the PC screen in front of the participant (black font, white background, middle of the screen). The person then listened to the sentence again, while also looking at it on the screen. The sentence that required such visual feedback remained in the database of the training sentences and was presented again at the end of the session. The training continued until all 148 sentences were repeated correctly without visual feedback, and the information about the required number of repetitions was stored for each person. The feedback was determined to be visual as opposed to auditory. In the future the authors wish to apply the same, albeit adjusted, testing (and training) procedures in participants with hearing deficits.

##### Selection of the speech stimuli for each stage of the experiment

At the beginning of the experiment, a MATLAB algorithm randomly selected two different lists of 10 HINT sentences for each of the 6 test conditions (3 before training and 3 after training) for each person individually. Another set of 148 sentences were used for the Training session. One list was saved for the Practice session.

### Ethics

The study was performed in accordance with all the relevant international and domestic guidelines and regulations, including the Declaration of Helsinki from 2013. Informed consent was obtained from each participant and they were compensated for participation. The experiment was approved by the Institutional Review Board of the School of Psychology of the Reichman University (formerly IDC), Herzliya.

### Data analysis

In the first step of the data analysis, we checked whether applying parametric tests to the data is valid. A Saphiro-Wilk test for normality revealed normal distribution of all participants’ scores in 6 tests (2 sessions × 3 conditions), the Mauchly Test of Sphericity was non-significant indicating equal variances of the differences between the factor levels, and no outliers were identified according to the 3 IQR’s rule. A repeated measures ANOVA was performed, with two within-subject factors: (a) Condition (Audio Only, Audio-Tactile matching, Audio-Tactile non-matching) and (b) Session (before and after Training) as well as one between-subject factor: (c) Group (Group 1 trained with the *audio-tactile matching* speech input vs Group 2 trained with the *audio only* speech input). Participants’ scores (SRT) in speech-in-noise understanding tasks were the dependent variable. The ANOVA was followed by a series of planned paired t-tests to assess the improvement from session 1 to session 2 (i.e. before vs after training) in speech-in-noise understanding in the three speech conditions separately, to compare the amount of improvement between test conditions, and to compare the scores between conditions within sessions. Independent-sample t-tests were applied to compare the scores in the two groups with one another, to test the order effect of the ATm and ATnm test conditions, and to compare the mean SRT values at which both groups were trained. To compare the number of repetitions needed for the participant to complete the training (i.e. to repeat all 148 sentences correctly) we applied a non-parametric Mann–Whitney *U* test (Shapiro–Wilk test of normality, p = 0.001). Since the scores for all speech tests were normally distributed and the Deviation of Linearity was > 0.05, we applied r-Pearson correlation analysis to: (a) asses the relationship between the scores in subsequent speech tests in session 1 (before training) and the amount of improvement from session 1 to session 2 in each of them (in all participants, as all of them improved), (b) the amount of benefit of adding matching vibrations as compared to the scores in the audio only condition (only in participants that had better SRT scores in the audio-tactile condition, i.e. 28 people before training, and 32 people after). Furthermore, we applied a correlation analysis to assess possible relationships between outcomes of the speech tests and several measures of the English language background of the participants. We tested whether the following factors might be related to some of the variability in participants’ performance in the speech tests: years of studying English and the average exposure to/communication in English everyday (mean of both at work/university and outside). Since the distribution of some variables was not normal (Shapiro–Wilk test, p < 0.05), we applied the non-parametric Spearman Rho approach. Furthermore, independent-sample t-tests were applied to compare SRTs in subsequent test conditions between participants that started learning to speak English before age 7 (early learners; N = 17) and after 7 (late learners; N = 17) (age 7 years was the median), as well as between participants that self-reported their English language abilities as ≤ 4.17 (the median, on a scale 1–5, N = 20), and those reported their skills as being above 4.17 (N = 20). SPSS (IBM Statistics 20) software was used to perform all the statistical analyses. GPower 3.1 software was applied for post-hoc assessment of the effect size (Cohen’s d for t-tests and Cohen’s f for F-tests) and power for each test. Due to the novelty of the study, there was no existing data to refer to in order to estimate the minimum sample size or the expected effect size a priori. In our previous study, though^31^ we used the same set-up but only two study conditions (*audio only* and *audio-tactile matching*) and showed a significant difference between the scores for the two conditions with a sample size of 12.

## Results

### Descriptive statistics

**Figure 3:**
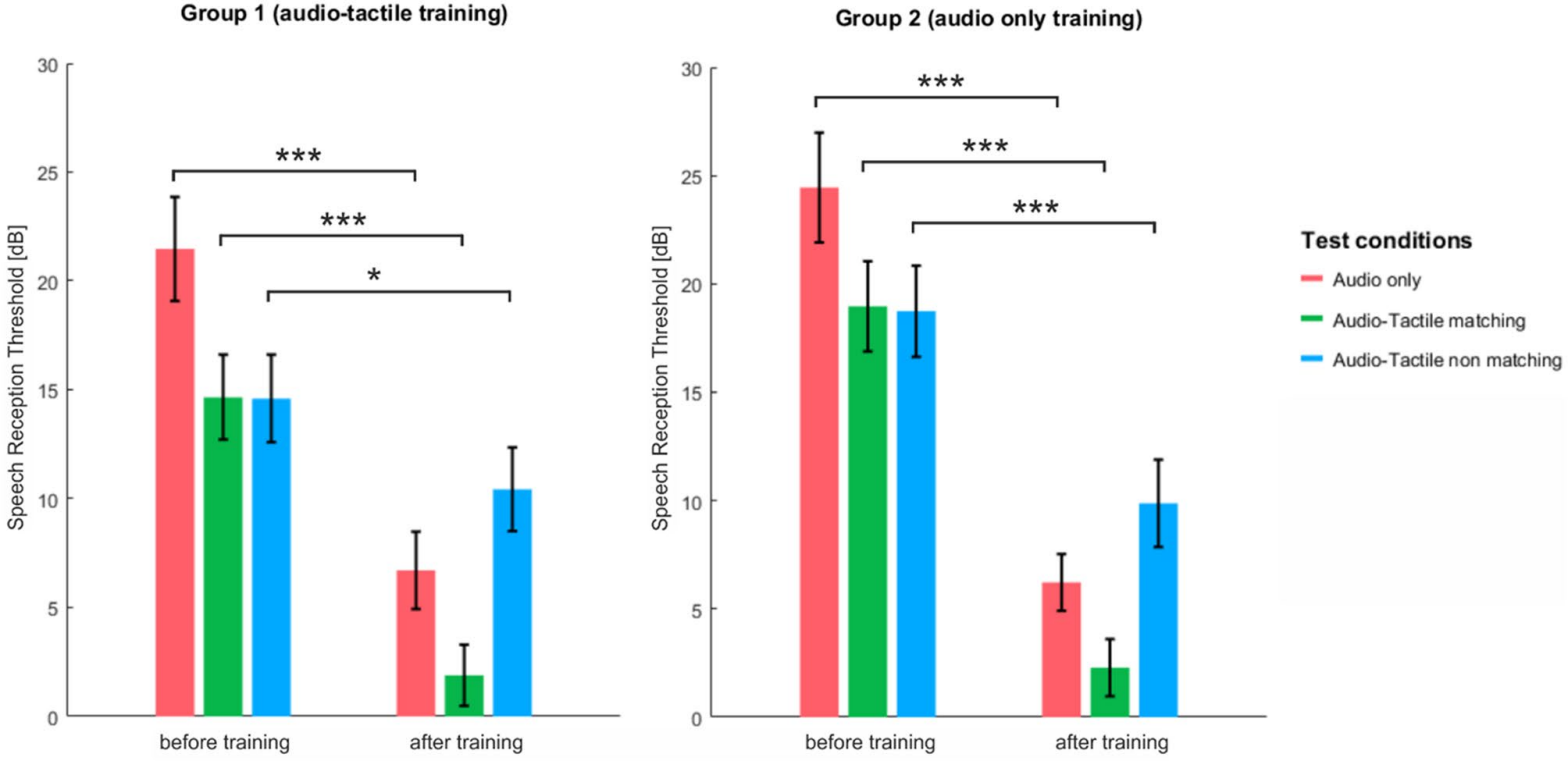
Speech reception thresholds in three test conditions before and after training in two groups separately; bars correspond to standard errors of the mean (Bonferroni, *indicates p < 0.017, **indicates p < 0.003, ***indicates p < 0.0003).

**Figure 4:**
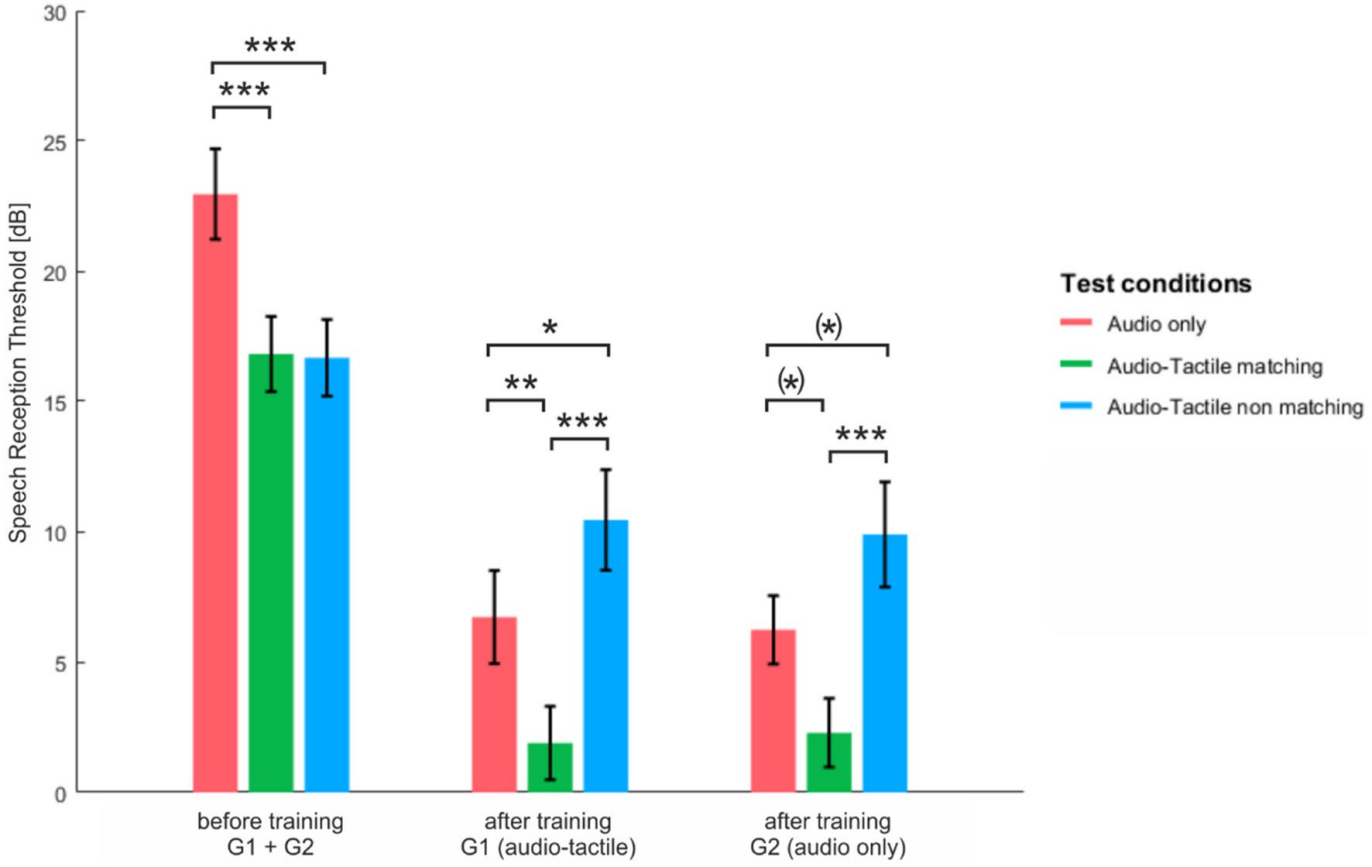
Speech reception thresholds in two sessions separately; bars correspond to standard errors of the mean; p values were Bonferroni corrected, *indicates p < 0.017, **indicates p < 0.003, ***indicates p < 0.0003.

We show in Table 2 and in Figs. 3 and 4 mean group results in all subsequent speech tests, both before and after the training session. It can be seen that the SRT values in both sessions as well as the pattern of improvement in all tests were quite similar in both groups of participants.

**Table 2:** SRT values in subsequent speech-understanding tests in both groups together and in each group separately.

Session	1 (before training)			2 (after training)		
Test	Audio only	Audio Tactile matching	Audio Tactile nonMatching	Audio only	Audio tactile matching	Audio tactile nonMatching
Both groups together	M = 22.96 (SD = 10.98)	M = 16.8 (SD = 9.15)	M = 16.67 (SD = 9.3)	M = 6.47 (SD = 6.9)	M = 2.09 (SD = 6)	M = 10.16 (SD = 8.7)
Group 1 (Audio tactile training)	M = 21.46 (SD = 10.68)	M = 14.66 (SD = 8.86)	M = 14.6 (SD = 8.96)	M = 6.71 (SD = 7.96)	M = 1.89 (SD = 6.28)	M = 10.44 (SD = 8.62)
Group 2 (audio only training	M = 24.44 (SD = 11.34)	M = 18.96 (SD = 9.31)	M = 18.73 (SD = 9.42)	M = 6.22 (SD = 5.85)	M = 2.28 (SD = 5.89)	M = 9.88 (SD = 9.02)

*M* mean, *SD* standard deviation.

### Results

#### Repeated-measures ANOVA

ANOVA revealed a statistically significant main effect of session (session 1 before training vs session 2 after training) [F(1,38) = 218.066 (p < 0.001, effect size = 0.85, power = 1)], and a statistically significant main effect of test condition (audio only, audio-tactile matching, audio-tactile non-matching) [F(2,37) = 18.599 (p < 0.001, effect size = 0.50, power = 1)]. Two interaction effects were revealed. The first one was an interaction effect between *Session* and *Condition* and was statistically significant [F(2,37) = 22.59, p < 0.001, effect size = 0.55, power = 1], and the second, between *Session* and *Group* [F(1,38) = 5.56, p = 0.024, effect size = 0.128, power = 0.63, adjusted alpha 0.05/12 = 0.004] was not statistically significant.

#### Within-group effects: improvement (from session 1 to session 2) in SRT in each condition

To assess the amount of improvement after training, we first combined the scores of the two groups together (N = 40). We interpret p-values as significant when they were below the Bonferroni-corrected alpha value of alpha = 0.05/3 = 0.017. We showed that the participants significantly improved from Session 1 to Session 2 in all three conditions : in the Audio Only (A) condition, from mean 22.96 ± 10 dB to mean 6.47 ± 6.9 dB [t(39) = 11.72, p < 0.001, effect size = 1.851, power = 1], in the Audio Tactile matching (ATm) condition from mean 16.8 ± 9.15 dB to mean 2.09 ± 6 dB [t(39) = 11.9, p < 0.001, effect size = 1.82, power = 1], in the Audio Tactile nonMatching (ATnm) condition from mean 16.67 ± 9.3 dB to mean 10.16 ± 8.7 dB, [t(39) = 5.46, p < 0.001, effect size = 0.86, power = 0.99]. The amount of improvement in A (by 16.5 ± 8.9 dB) and in ATm (by 14.7 ± 7.8 dB) were not statistically significantly different (p = 0.29) and the amount of improvement in the ATnm condition was statistically lower than for the two remaining conditions [t(39) = 6.17, p < 0.001 when compared to A; t(39) = 5.5, p < 0.001 when compared to ATm]. Both groups showed a similar pattern of improvement for all speech test conditions. Group 1 improved in the A condition from 21.46 ± 10.68 to 6.71 ± 7.96 [by 14.76 ± 7.78; t(19) = 8.48, p < 0.001, effect size = 1.897, power = 1], in the ATm condition from 14.66 ± 8.68 to 1.89 ± 6.28 [by 12.78 ± 6.05; t(19) = 9.45, p < 0.001, effect size = 2.114, power = 1], and in the ATnm condition from 14.60 ± 8.96 to 10.44 ± 8.62 [by 4.17 ± 7.0; t(19) = 2.66, p = 0.016, effect size = 0.594, power = 0.819] (Fig. 3). Group 2 improved in the A condition from 24.44 ± 11.34 to 6.22 ± 5.85 [by 18.2 ± 9.78; t(19) = 8.33, p < 0.001, effect size = 1.865, power = 1], in the ATm condition from 18.96 ± 9.31 to 2.28 ± 5.89 [by 16.68 ± 9.0; t(19) = 8.28, p < 0.001, effect size = 1.854, power = 1], and in the ATnm condition from 18.73 ± 9.42 to 9.88 ± 9.02 [by 8.85 ± 7.5; t(19) = 5.28, p < 0.001, effect size = 1.182, power = 0.99] (Fig. 3). The effect size of the improvement in the audio-tactile non-matching test condition was larger in Group 2 than in Group 1 (1.182 vs 0.594, respectively; although the degree of improvement was not statistically significantly different in the direct comparison between groups, t(38) = − 2.04, p = 0.048, Bonferroni-corrected alpha = 0.017).

#### Within-group effects: comparisons between SRTs in subsequent speech test conditions in each session

With the scores of both groups combined (N = 40; Fig. 4) we showed that in Session 1 mean group SRT values in the *Audio Tactile matching* condition and in the *Audio Tactile nonmatching* condition were both statistically significantly lower (indicating better performance) than those obtained in the *Audio only* condition [t(39) = 4.53, p < 0.001, effect size = 0.71, power = 0.99, and t(39) = 4.8, p < 0.001, effect size = 0.76, power = 0.99, respectively). At the same time, the scores in the ATm and the ATnm test conditions were found not to be statistically significantly different from one another (p = 0.914). In addition, no order effect was revealed for these two conditions, neither before or after training (p > 0.05). In Group 1 the pattern of the results was very similar to the combined group results. The difference in SRT values was statistically significant between the A and the ATm speech test scores [t(19) = 3.52, p = 0.002, effect size = 0.630, power = 0.86] and between the A and the ATnm test scores [t(19) = 4.54, p < 0.001, effect size = 0.589, power = 0.81], whereas scores in the two audio-tactile tests were not statistically different (p = 0.97). In Group 2 as well SRT values were statistically significantly different for the A and the ATm speech test conditions [t(19) = 2.82, p = 0.011, effect size = 0.786, power = 0.96] and for the A and the ATnm speech tests [t(19) = 2.63, p = 0.016, effect size = 1.01, power = 0.99), and not different for the two multisensory tests (p = 0.914).

In Session 2 the mean group SRT for all 40 participants was lowest (indicating best performance) for the *Audio Tactile matching* test condition and highest for the *Audio Tactile nonmatching* test condition (poorest performance) [when compared with one another, t(39) = 8.22, p < 0.001, effect size = 1.296, power = 0.99]. The scores obtained in the *Audio only* test condition were significantly higher than those reported for the ATm test [t(39) = 8.2, p < 0.001, effect size = 0.709, power = 0.92) and significantly lower than those shown for the ATnm test [t(39) = 3.9, p < 0.001, effect size = 0.616, power = 0.84). We also performed the same analysis for both groups separately. In Group 1 we found statistically significant differences between all test conditions [A vs ATm, t(19) = 3.97, p = 0.001, effect size = 0.567 , power = 0.79; A vs ATnm, t(19) = 3.3, p = 0.004, effect size = 0.532, power = 0.74; ATm vs ATnm, t(19) = 6.7, p = 0.000, effect size = 1.117, power = 0.99). In Group 2 the SRT scores in the ATm an ATnm tests were statistically significantly different [t(19) = 5.0, p < 0.001, effect size = 1.497, power = 0.99], while the difference between the scores in the ATm and the A tests [t(19) = 2.54, p = 0.02, effect size = 0.887, power = 0.98), as well as between A and ATnm showed a trend for significance [t(19) = 2.4, p = 0.028, effect size = 0.737, power = 0.94). For each session, we interpreted p-values as significant when they were below the Bonferroni-corrected alpha value of alpha = 0.05/3 = 0.017. These results have been presented in Fig. 4.

#### Between-group effects

When mean scores obtained by the two study groups were compared with one another in two sessions and in each test condition separately (Group 1 vs Group 2, independent t-tests), we found no statistically significant differences (see *Supplementary Materials*, Tables 1–2).

#### Training

Participants in Group 1 that trained with multisensory audio-tactile speech inputs needed on average 359.8 ± 132.1 repetitions of the sentences to accurately repeat all 148 of them (at a mean SRT of 14.7 ± 8.7 dB). Participants in Group 2 that trained with unisensory auditory speech inputs needed on average 351.6 ± 80 repetitions (at a mean SRT of 23.9 ± 11.8 dB). Mean SRT values at which the groups were trained were statistically significantly different between the groups (t = -3.2, p = 0.003). The required number of sentence repetitions in both study groups was not statistically significantly different (Mann–Whitney U = 184, p = 0.665).

#### Correlation analyses

**Figure 5:**
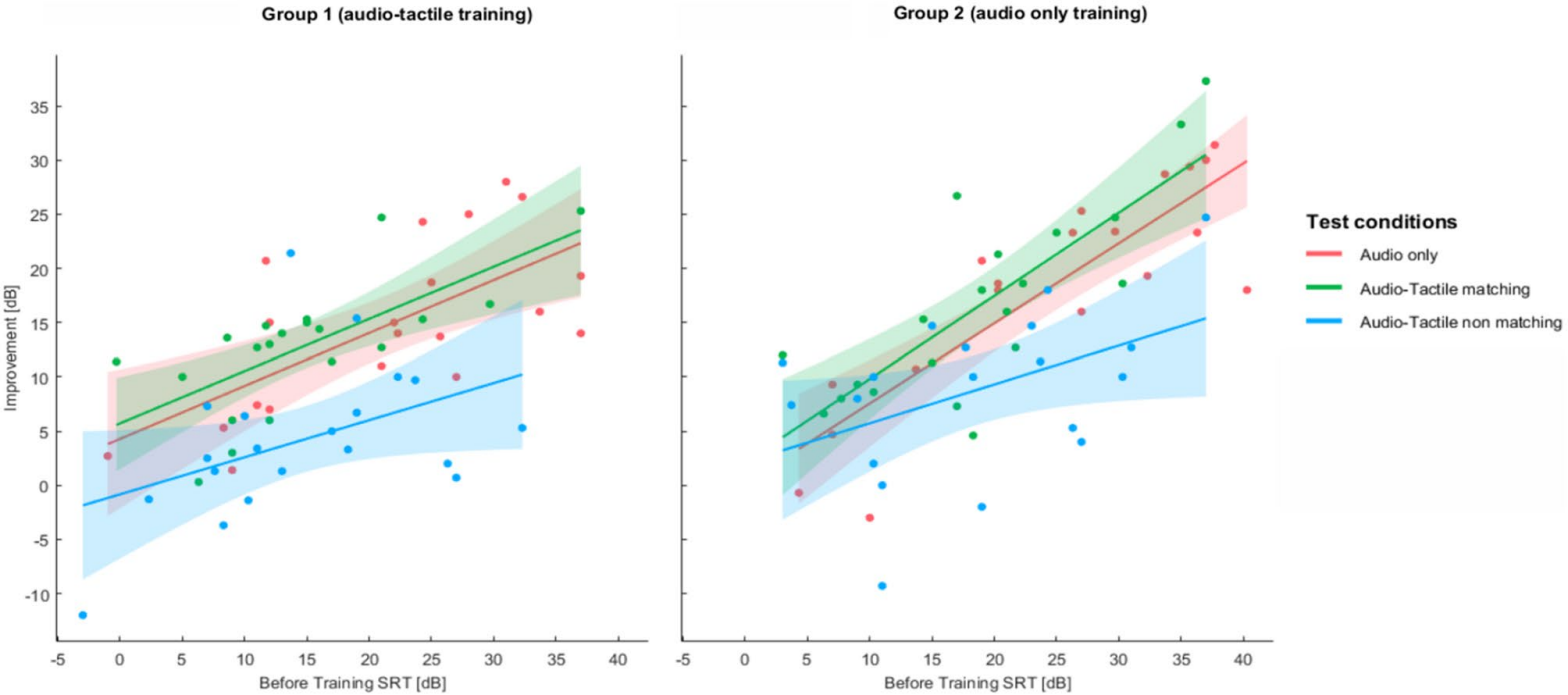
Scatterplots showing a positive relationship between SRT values obtained in three tests before training and the amount of improvement in each of them; shadowing corresponds to a 95% confidence interval.

We found statistically significant correlations between participants’ scores in subsequent speech tests and the amount of the corresponding improvement in each of them (Fig. 5). The found statistical values were the following: (a) SRT for A1 and improvement in A (both groups together, R = 0.78, p < 0.001; Group 1, R = 0.67, p = 0.001; Group 2, R = 0.86, p < 0.001), b) SRT for ATm1 and improvement in ATm (both groups together, R = 0.76, p < 0.001; Group 1, R = 0.69, p = 0.001; Group 2, R = 0.79, p < 0.001), SRT for ATnm and improvement in ATnm (both groups together, R = 0.48, p = 0.002; Group 1, R = 0.44, p = 0.054; Group 2, R = 0.45, p = 0.047). As the data shows and it can be seen in Fig. 5, the R-Pearson correlation was found significant for all speech tests, but was least significant for the ATnm condition.

In addition, we found that scores in the *auditory only* speech test positively correlated with the amount of improvement yielded by adding matching tactile vibrations (ATm1-A1) (R = 0.57, p = 0.002), but only before training. The result indicates that the multisensory benefit was most profound when the unisensory performance was poorest. After training, the relationship was not statistically significant (p < 0.05).

#### Additional results using English language background information

With respect to the additional background information that we collected about the participants, we showed that participants who started learning to speak English at age < 7 had lower SRT values (better performance) by mean 2–6 dB in three speech tests before training than those that started learning after age 7 but the difference in scores was not statistically significant at Bonferroni-corrected alpha value of 0.05/3 = 0.017. At the same time, however, years of studying English were found to correlate with the SRT scores in the *Audio only* test (rho = − 0.27, p = 0.021) and the *Audio-Tactile nonmatching* test (rho = − 0.52, p = 0.001) before training, but not with the ATm speech test scores (rho = − 0.197, p = 0.22) (N = 40). This result indicates that more years of studying were related to lower (better) SRT values. In addition, higher average exposure (more frequent communication) to English was found to correlate with lower (better) SRT scores in the *Audio-tactile nonmatching* speech test before training (rho = − 0.362, p = 0.022). We also found an effect of self-rated English abilities, in that the participants who rated their skills as higher (above the median 4.17 on scale 1–5) as opposed to lower had consistently lower (better) SRTs in the speech tests. The effect was, however, only statistically significant for the *Audio only* test condition [t(38) = 2.53, p = 0.016, effect size = 0.8, power = 0.8; Bonferroni-corrected alpha value 0.017). More detailed results of these analyses are presented in *Supplementary Materials* (Table 3–5)*.*

## Discussion

### Summary of the results

In the current experiment we replicated in a larger group of 40 participants our previous proof-of-concept study where we also showed enhanced speech-in-noise understanding (by mean 4–6 dB) when auditory speech signals were complemented with matching low-frequency tactile vibrations on fingertips^31^. As an addition to the previous study where no training was applied, we also used here two training types to test whether speech comprehension in noise can improve further. We found profound and comparable degree of improvement in performance, both after unisensory auditory training and after multisensory audio-tactile training. The effect generalized to novel sentences and both unisensory and multisensory test conditions. In addition, the amount of the improvement shown in both *audio only* and *audio-tactile matching* test conditions was similar and exceeded 10 dB (mean 14–16 dB) in SNR/SRT. Such a decrease in the speech in noise reception threshold, is profound, as a 10 dB difference represents doubling of the perceived loudness^62^. Furthermore, participants trained with multisensory inputs (group 1) that were presented with sentences at a lower SNR (harder) than the other group needed a similar number of sentence repetitions to complete the training. This might be an indication that adding vibrotactile inputs can make the learning process easier, although further studies are necessary. The participants were furthermore tested on understanding sentences when the vibrations on the fingertips corresponded to a different sentence than the one presented as audio (i.e., they were non-congruent), both before and after training. For this test condition before training, we found a similar degree of benefit over *audio only* as when matching tactile inputs were used, and lower scores than in the *audio only* test condition following both types of training. This outcome, as well as the reported improvement in all testing conditions shows that the applied training (both uni- and multisensory) possibly removes some level of difficulty of sound perception per se, and hence the participants might be able after training to use the speech input delivered via vibrotactile stimulation more properly. We discuss below a number of theoretical aspects of the reported results, as well the potential implications of our study for basic science and rehabilitation.

### Speech training generalization and unisensory vs multisensory training

There have been numerous works showing that understanding speech in suboptimal acoustic conditions, such as e.g., speech in noise, noise-vocoded speech, time-compressed speech, can be trained. At the same time, however, the reported transfer of the training effects to novel speech inputs or contexts has been found to be limited^63–66^. In that respect, our findings are novel, as we show not only significant benefits for speech understanding (> 10 dB in SRT) but also generalization of both training types to all three testing conditions (*audio only, audio-tactile matching, audio-tactile nonmatching*). We therefore report a transfer of improvement from a multisensory to a unisensory context, as well as in the opposite direction. Each time the effect was shown for completely novel sentences. A similar idea of multisensory training boosting unisensory auditory and visual speech perception, was shown by Bernstein and colleagues^20^ and by Eberhard and colleagues^25^, respectively (although the applied language tasks were more basic than repeating whole sentences). We suggest that this robust and rapid improvement can have a number of practical applications, including in sensory rehabilitation, which we discuss further in text. We showed that both training types, the unisensory auditory training and the multisensory audio-tactile training, provided similar level of improvement in terms of the SNR change. This is probably due to the fact that the auditory input was present in both of them and thus practiced to a similar extent. Indeed, rapid perceptual learning and adaptation have been shown through repeated exposure for various acoustic contexts and distortions of the auditory signal, including natural speech presented in background noise, as well synthetically manipulated vocoded/time-compressed speech^2,3,55,63,66^. This also explains why we saw some improvement in speech understanding in the control test condition, i.e., when the auditory sentences were paired with vibro-tactile inputs corresponding to a different sentence than the one presented through audition (ATnm). The degree of improvement was, nevertheless, far less robust in that test condition, as compared to the trained ATm and A conditions (approx. 6 dB vs 14–16 dB in SRT). Notably, the effect size of the improvement in the non-congruent audio-tactile condition was also smaller in the group trained with the matching audio-tactile inputs. This, as well as the fact that this group showed much more significant benefit of adding congruent vibrations to the auditory speech input after training, points to the specific effect of the applied training type. At the same time, however, adding tactile inputs might have facilitated the learning process, as the same degree of improvement (by mean > 10 dB) was achieved by Group 1, as compared to Group 2 (trained with audio inputs only), at harder SNR values. This finding is in line with some existing studies suggesting that learning and memory of sensory/cognitive experiences is more efficient when the trained inputs are multisensory, such as in patients after stroke^19^ or in hemianopia (i.e., with loss of vision in only one part of the visual field and/or one eye^20^]. In addition, we showed, using correlation analysis, that the improvement after training was reversely proportional to the initial test outcomes (as was expected and often reported in literature). It remains to be studied, whether a longer training session or training without feedback but with a fixed number of sentence presentations would be more appropriate to examine the benefit of one training approach over the other.

### Multisensory enhancement and audio-tactile speech input

We show in our study that 70% participants before and 80% participants after both types of training achieved best scores in the test condition that combined an auditory input with a matching tactile input. We believe that this finding might exemplify the inverse effectiveness rule of multisensory integration which predicts that multisensory enhancement, i.e., the benefit of adding information through an additional sensory channel, is especially profound for low signal-to-noise conditions (but see the possible role of order in the *Limitations of the study* paragraph)^52^. To further support this claim, we also showed, using correlation analysis, that the benefit for speech perception (SRT values) of adding matching tactile vibrations on fingertips to the degraded auditory signal increased before training, the poorer the person performed in the *audio only* test condition. This effect was not maintained after training, when auditory speech recognition already much improved. Indeed, our experimental procedure was specifically designed to benefit from the inverse effectiveness principle^5,67^. In our study the auditory speech signal was new to the study participants, degraded (noise-vocoded), presented against background noise and in their non-native language. All these manipulations lead to a low signal to noise context, and deemed the auditory input less reliable, thus increasing the chance that a reliable tactile input will improve performance.

To deliver tactile inputs, we developed our own audio-to-touch SSD. In the literature it has been suggested that SSDs can advance the benefits of providing a multisensory learning environment, since they convey input from one modality via another, in a way that is specifically tailored to the neuronal computations that are characteristic of the original sensory modality^41–47^. The delivered tactile inputs were complementary to the auditory speech. They also maintained features typical of the auditory modality, in that tactile vibrations are also a periodic signal that can fluctuate in frequency (and intensity). At the same time, the applied frequency range of the inputs was detectable by the tactile Pacinian cells that are most densely represented on the fingertips (as compared to other body parts) and most sensitive for encoding frequencies in the range of 150–300 Hz (and up to 700–1000 Hz)^68^. Specifically, the tactile vibration that was provided on fingertips of our participants was the low-frequency part of the speech signal, including the extracted fundamental frequency. The assumption that this specific input might help with speech perception was based on findings in healthy individuals (and hearing impaired, as discussed further in the text) that have been shown to benefit in their speech-in-noise understanding from addition of low-frequency sounds, which carry pitch information and thus help segregate auditory streams, including discriminating between speakers^29,32^.

We believe that the specific combination of audition and touch, the two senses that are indeed quite similar as they both encode vibrations through mechanoreceptors, made this multisensory context in our study especially intuitive. This might have contributed to the best of scores in speech-in-noise understanding in the test combining both congruent inputs, after both types of training, as well facilitate the learning process. Notably, the reported rapid improvement is in contrast to other SSD solutions that were based on more complex algorithms (e.g. translating visual images to sounds changing in pitch and timbre) and required hours of training to yield benefits^47–49^.

To the best our knowledge, there have only been several research works thus far that have studied improvement in speech understanding by adding speech signals through touch, in normal hearing subjects with training^29^ and in patients with hearing loss without any training^30,69^. In Fletcher’s work 8 participants showed after unisensory auditory training a mean improvement of 10% in understanding of CI-simulated sentences in noise when the participants also received concurrent low-frequency tactile stimulation on the fingertips. Before training the difference was at 5% and was not statistically significant. The authors tested speech comprehension at fixed SRTs, which were nevertheless individually established using an adaptive procedure^61^. At the same time, however, the participants were trained during 3 days at arbitrarily established harder SRT values, whereas we used the same individual SRT values during the whole training session (established separately for the *auditory only* and the *audio-tactile* training session). Due to these methodological differences, we cannot directly compare our findings with those reported by^29^. Nevertheless, we would like to note that the authors failed to report a significant benefit of adding tactile vibrations before training, as opposed to what we showed for our set-up in our previous and current work. In addition, the authors reported that speech in noise understanding with added touch improved by 10% due to training (61% to 71% of recognized keywords) but at the same time did not discuss this finding at all. Meanwhile, Huang and colleagues used a similar set-up to study speech in noise understanding in a group of 10 users of cochlear implants. The authors reported a 2.2 dB improvement in sentence understanding when participants were provided with additional low-frequency speech inputs, including f0, on their fingertips. The 2.2 dB improvement is less profound than the 4–6 dB multisensory improvement reported in our previous and current work. The underlying reason might be that the patients did not undergo any training, which might be specifically needed in the patient population due to the high cognitive demand required for speech in noise understanding^5,10,30^. While both^29^ and^30^ applied an experimental approach quite similar to ours, Perotta and colleagues used a wristband with linear actuators that delivered vibrations at a constant frequency and showed that (N = 18) hard-of-hearing patients can learn to discriminate word pairs based on patterns of vibrations. All these studies show that there is indeed a potential in using specifically prepared tactile information to boost speech understanding. At the same time, our current study is novel in that we collected data in a significantly larger group of healthy individuals (N = 40). In addition, we designed a control unisensory auditory training condition to estimate the effect of repeated exposure to the auditory input per se. We also added a control multisensory condition, with tactile speech frequencies that do not match the presented auditory sentence, to further investigate the role of touch. Furthermore, the participants of our study were non-native English speakers, the implications of which we discuss in subsequent paragraphs. We can only speculate that the differences in the outcomes in our study and the ones by^29^ and^30^ were also related to the fact that these authors tested native speakers of English.

### The revised plasticity theory

The level of multisensory benefit that we report in our study, both before and after training (4–6 dB) is comparable or higher to that reported when auditory speech in noise is complemented with cues from lip/speech reading^70–73^. Since we have not tested audio-visual speech understanding in the current experiment we cannot, however, compare the outcomes of the two multisensory speech contexts directly. This idea is nonetheless worth further investigation, as synchronous audio-visual speech information is what we as humans are exposed to from the very early years of development and throughout our lifetimes^9^, as opposed to the audio-tactile speech input that is an utterly novel experience, at least for the tested healthy individuals (we talk about the specific case of the visually impaired further in text). We therefore argue that through a well-designed intervention one can establish in adulthood a new coupling between a given computation and an atypical sensory modality that has never been used for encoding that type of information before. We also show that this coupling can be improved through training, as all participants in the current experiment significantly improved in that test condition. These findings might provide further support for our new conceptualization of critical/sensitive periods in development, as presented in our recent review paper^22^. We suggest that brain plasticity, which indeed spontaneously decreases with age, can be reignited across the lifespan, even with no exposure to certain unisensory or multisensory experiences during childhood. Several studies from our lab and other research groups, mainly involving patients with congenital blindness, as well as the current study, point in that direction (e.g. by showing that congenitally blind participants can use hierarchically organized brain visual networks for recognition of faces, body shapes, letters, etc. with the stimuli conveyed through audio-visual SSDs)^21,74,75^. A similar line of research (although not with an SSD and for another pair of senses) was also reported by Ernst and colleagues where participants learned to integrate signals from vision and touch (luminance and stiffness) and after training showed increased discrimination thresholds (poorer performance) for non-congruent vs congruent pairs of stimuli^76^.

### The control tactile condition

In our experiment we introduced a control audio-tactile condition with vibrations not corresponding to the auditory sentence, to test how it would affect speech-in noise understanding. Adding this control condition is novel and was not studied thus far in works dedicated to tactile speech processing^29,30,69^. In the literature, it has often been shown that while a congruent input from two sensory modalities can improve performance through cross-modal interactions^23,24^, non-matching or distracting information in one sensory modality can impair performance^5,70,77–79^. Indeed, we show here that after training both groups of participants had poorest scores in the *audio-tactile non-matching* test condition and for both groups the improvement in this speech test was least significant. At the same time, however, the scores before training were almost the same for both conditions combining an auditory and a tactile input, i.e. for both congruent and non-congruent pairs. The revealed mean SRT values for both of them were also significantly better than those reported for the *audio only* test condition. This indicates a possible non-specific multisensory effect before training, i.e., during the period of familiarization with the audio-tactile study set-up. The non-specific tactile input might possibly be helping the participants to keep their attention on the task. Although the vibrations in the control condition were not congruent with the speech signal, they still resembled the target sentence more than the background noise and thus helped the participants focus on the target input. The data suggests that within a short time participants learned to ignore the non-matching vibration, which led to the poorest scores in this test condition after training. Alternatively, and as already suggested above, participation in training may have removed some level of difficulty of sound perception, which made it easier for the participants to properly use the speech information present in vibrotactile stimulation. Therefore, we believe that the results of the post-training test session might actually be more representative of the benefit of adding speech-related information through touch for speech perception [see Ernst and colleagues for a similar effect of training congruent vs non-congruent visuo-tactile pairs of stimuli^76^]. In addition, the fact that one group trained with congruent tactile inputs seems to have resulted in their lesser improvement in the *audio-tactile nonmatching* condition as well as poorer audio-tactile nonmatching scores after training (as compared to the group trained with unisensory auditory inputs). The reason might be related to the fact that the ATnm test condition might have actually been the hardest in terms of the required cognitive resources, including selective attention, and inhibition from distraction^79^. In order to elucidate the effects of the multisensory congruency effect further, research involving controlled selective attention of the participants is needed. One can speculate that a training session of focusing attention on the inputs from a single sensory modality might both reduce the distracting effects of the non-matching tactile input (when focused on the auditory aspect) and improve the benefit of adding matching vibrations further (when focused on the matching tactile input).

### Non-native speakers of English

The reason why we focused on non-native English speakers in our study was to further benefit from the inverse effectiveness rule of multisensory integration requiring low SNR between senses. At the same time, we made sure that the participants were fluent in English and felt comfortable with the experimental setting. Non-native listeners have been shown to perform much poorer in speech-in-noise tasks than native English speakers. The reduced performance has been attributed to a number of factors, such as language proficiency, degree of exposure to the foreign language and age of language acquisition^10,27,55–57^. In the current experiment we used the HINT sentence database consisting of sentences with semantic content which are to some extent predictable^60^. Therefore, we assumed (and actually witnessed during the study) that the participants would apply their high-level knowledge to improve perception and predict/guess the upcoming language information. We performed several additional analyses to test, whether the English language background of the participants would translate to their speech scores before training. Indeed, we showed that a longer time of studying English, more exposure to it in both professional and leisurely contexts, as well as better self-rated skills in English were related to better initial scores in speech comprehension through audition (A1). More years of studying and higher everyday exposure to English translated also to better scores in the *audio-tactile nonmatching* test condition (ATnm1). As mentioned before, this effect might be related to the fact that the noncongruent multisensory task requires additional cognitive resources, which might have been trained in the foreign-language contexts throughout life. In addition, we found that the participants that started learning English at an earlier age (< 7 years of age) had better scores in all speech-in-noise understanding tasks before training, although this effect was not statistically significant. We therefore provide no strong evidence for the existence of “critical periods” in development for the second language acquisition^56^. This effect might possibly occur for language acquisition at ages below 3 (e.g. vs in adulthood), while only 4 of our participants started learning English that early in life.

For non-native English speakers it has also been shown that in suboptimal speech contexts they might more readily attend to inputs from additional modalities, such as vision. This has been observed in contexts, such as the McGurk effect during which non-congruent lip-reading cues affect auditory perception, as well as when congruent gestures are present to support speech perception^10,58^. It has been suggested that this higher reliance on visual cues might stem from the linguistic challenge that non-native listeners experience, but at the same time does not have to translate to improved performance in speech understanding^27^. To the best of our knowledge no studies exist that investigated audio-tactile speech contexts, where the same testing and training set-up was applied in native and non-native English speakers. We nevertheless speculate that native English speakers will also benefit from our solution, but due to the better language skills might need to be exposed to poorer SNRs for the multisensory benefit to be detected. Further research on this is required.

### Applications of our set-up and audio-tactile SSD for sensory rehabilitation

Thus far, the results of our previous and current studies show that our set-up can assist healthy individuals in improving their understanding of distorted speech in background noise. This already indicates the possible general application of our solution in learning foreign languages. In addition, our technology and the tactile feedback can also potentially improve understanding speech over the phone, as well as appreciation of music.

In the current experiment the trained speech signal was distorted (noise-vocoded) in order to recreate the experience of speech perception through a cochlear implant. This was done, as we are planning to test and train audio(-tactile) speech perception in patients with hearing deficits in our next study. We believe that our set-up holds promise for rehabilitation in this population as these patients specifically struggle with understanding speech in noise, despite their good performance in quiet environments^15–17^. The underlying reason for this disadvantage is that the degraded auditory input that the patients are exposed to is deprived of the temporal fine structure information (the rapid oscillations close to the fundamental frequency, f0/pitch)^32^ which is the basic cue for speech stream segregation and discrimination among speakers^80^. In sensorineural hearing loss this information is impaired or lacking due to the damage to the inner ear, whereas in CI-users it is due to the limitations of the algorithms applied for speech coding^81,82^. Some evidence of the benefit of adding low-frequency speech cues for speech understanding was shown for patients with partial deafness (high-frequency sensorineural hearing loss) who use a partially inserted CI electrode array combined with low-frequency acoustic signal delivered naturally or via a hearing aid in the same ear. These patients consistently show superior performance in speech-in-noise understanding, when compared to profoundly deaf users of CIs^33,80,83,84^. In addition, Huang and collegues^30^ already showed improved speech-in-noise understanding in a group of CI users when sentences were accompanied with low-frequency vibrations corresponding to the extracted low frequency sounds from the speech input, with no prior training. Nevertheless, we believe that in the case of the hearing impaired, for whom speech-in-noise understanding requires more cognitive effort than in healthy individuals, training might actually be required to yield visible benefits. Indeed, users of cochlear implants have been found to improve following extensive audio-visual training sessions combining the restored auditory input with speech-reading or sign language^1,6,65,70,73,86,88^. As we show in the current study, speech understanding can successfully be trained (both through unisensory and multisensory inputs) using our set-up. In addition, with future hard-of-hearing candidates for cochlear implantation, we believe that both unisensory auditory (or maybe also unisensory tactile, though it has not been tested here) and multisensory audio-tactile training can be applied, with the aim of “preparing” the auditory cortex for future processing of the auditory input. We speculate that this can be achieved, based on a number of works from our lab which show that specialization of the sensory regions in the brain, such as the (classically termed as) visual or the auditory cortex, can emerge also following a specifically designed training regimen with inputs coming from another modality, as long as they preserve specific computational features (Task-Selective and Sensory-Independent (TSSI) brain organization)^22,85,87,89^.

Another interesting line of study by the Petrini group shows yet another possible implementation of our audio-tactile SSD set up. The research of the group shows that audio-haptic integration, and namely discrimination of object sizes by touching and/or listening to them is consistently less efficient in individuals with late onset visual impairments, as compared to the early-blind and the healthy population^59,77^. The authors explain that since touch and not vision is the dominant modality for performance in this specific task, early blind individuals are not affected. Their results also suggest that enhancement of perceptual functioning in remaining senses (i.e. in the visually impaired, audition and touch) is practice-dependent and thus related to the consistency of the experience. We therefore suggest that our set-up could be applied to train audio-tactile integration abilities in these individuals. Especially since audition and touch and their successful integration are what the blind population relies on (this would obviously require adjustments of the study paradigm, including e.g., using an auditory instead of visual feedback during the training session). It would be also interesting to see how the fact that part of this population uses Braille reading, which requires matching certain tactile inputs with language units, would be reflected in training outcomes using our audio-tactile SSD set up.

## Future directions of our research

We believe that development of assistive communication devices, both with and without tactile cues is especially needed in the time of the COVID19 pandemic which imposes numerous restrictions on live communication, including the limited access to visual speech cues from lip reading. The robust outcomes, that we have obtained after a relatively short and easy training session of < 1 h can make our set-up a valuable and effective tool for rehabilitative, professional, and educational settings. Although our set-up is already relatively small in size, as opposed to the more cumbersome solutions using tactile inputs that are available on the market^48,90^, our lab already started developing new minimal tactile devices that can provide vibrotactile stimulation also on other body parts, beyond the fingertips. Our aim is to design a set-up that will be wearable and therefore will assist with speech-in-noise comprehension (and sound source localization) in real-life scenarios. In addition, our current SSD is compatible with a 3 T MRI scanner. We have already collected functional magnetic resonance (fMRI) data in a group of participants performing the same tasks of unisensory and multisensory speech comprehension. The results can help uncover the brain mechanisms of speech-related audio-tactile binding and improvement, and may provide information on inter-subject variability in relation to the benefits of multisensory learning. This latter aspect, in turn, could further direct rehabilitation and training programs. To the best of our knowledge this would be the first attempt at elucidating neural correlates of understanding speech presented as a combined auditory and vibrotactile input.

## Limitations of the study

In the testing session the *auditory only* speech test (A) always preceded the audio-tactile test conditions (ATm, ATmn). We therefore cannot exclude some effect of order, with respect to the obtained benefits of the complementary tactile speech information. We nevertheless believe that if there was such an effect, it was minimal, especially after exposure to > 200 sentences before the post-training test session. We also found no order effect for the two multisensory audio-tactile test conditions, neither before nor after training (as we reported in the “Results” section). In addition, the current study only shows immediate outcomes of the applied training and thus further studies are needed to assess whether long-term training protocols can further improve and/or retain the effects of learning, and which components of the training best enable this goal. The long-term effects are especially crucial for efficient sensory rehabilitation.

## Supplementary Information

Supplementary Information.
