# Supplementary material for: Effects of training and using an audio-tactile sensory substitution device on speech-in-noise understanding
Source: Sci Rep. 2022 Feb 25;12:3206. doi: 10.1038/s41598-022-06855-8 (PMC8881456; doi:10.1038/s41598-022-06855-8)
Supplement: Supplementary file 1 — Supplementary Information. [file 41598_2022_6855_MOESM1_ESM.docx]

| **coTest** | **Group**  **(G1 – AudioTactile, G2 – Audio only)** | **N** | **Mean** | **Std. Deviation** | **Std. Error Mean** |
| --- | --- | --- | --- | --- | --- |
| A1 | G1 | 20 | 21.4650 | 10.68965 | 2.39028 |
|  | G2 | 20 | 24.4450 | 11.34625 | 2.53710 |
| ATm1 | G1 | 20 | 14.6650 | 8.68442 | 1.94190 |
|  | G2 | 20 | 18.9600 | 9.31684 | 2.08331 |
| ATnm1 | G1 | 20 | 14.6050 | 8.96029 | 2.00358 |
|  | G2 | 20 | 18.7300 | 9.42405 | 2.10728 |
| A2 | G1 | 20 | 6.7100 | 7.96234 | 1.78043 |
|  | G2 | 20 | 6.2250 | 5.85157 | 1.30845 |
| ATm2 | G1 | 20 | 1.8900 | 6.28205 | 1.40471 |
|  | G2 | 20 | 2.2850 | 5.89266 | 1.31764 |
| ATnm2 | G1 | 20 | 10.4400 | 8.62563 | 1.92875 |
|  | G2 | 20 | 9.8800 | 9.02561 | 2.01819 |

**Supplementary Materials**

**Table 1. Outcomes obtained in subsequent speech tests (SRT values), in all participants, with M and SD.**

**Table 2. Outcomes obtained in subsequent speech tests (SRT values) : the results of between-group comparisons (independent t-tests).**

|  | | Levene's Test for Equality of Variances | | t-test for Equality of Means | | | | | | |
| --- | --- | --- | --- | --- | --- | --- | --- | --- | --- | --- |
|  |  | F | Sig. | t | df | Sig. (2-tailed) | Mean Difference | Std. Error Difference | 95% Confidence Interval of the Difference | |
|  |  |  |  |  |  |  |  |  | Lower | Upper |
| A1 | Equal variances assumed | .119 | .733 | -.855 | 38 | .398 | -2.98000 | 3.48573 | -10.03649 | 4.07649 |
|  | Equal variances not assumed |  |  | -.855 | 37.866 | .398 | -2.98000 | 3.48573 | -10.03731 | 4.07731 |
| ATm1 | Equal variances assumed | .182 | .672 | -1.508 | 38 | .140 | -4.29500 | 2.84801 | -10.06049 | 1.47049 |
|  | Equal variances not assumed |  |  | -1.508 | 37.814 | .140 | -4.29500 | 2.84801 | -10.06142 | 1.47142 |
| ATnm1 | Equal variances assumed | .086 | .771 | -1.419 | 38 | .164 | -4.12500 | 2.90774 | -10.01142 | 1.76142 |
|  | Equal variances not assumed |  |  | -1.419 | 37.904 | .164 | -4.12500 | 2.90774 | -10.01191 | 1.76191 |
| A2 | Equal variances assumed | 1.519 | .225 | .220 | 38 | .827 | .48500 | 2.20952 | -3.98794 | 4.95794 |
|  | Equal variances not assumed |  |  | .220 | 34.889 | .828 | .48500 | 2.20952 | -4.00108 | 4.97108 |
| ATm2 | Equal variances assumed | .529 | .472 | -.205 | 38 | .839 | -.39500 | 1.92597 | -4.29393 | 3.50393 |
|  | Equal variances not assumed |  |  | -.205 | 37.845 | .839 | -.39500 | 1.92597 | -4.29445 | 3.50445 |
| ATnm2 | Equal variances assumed | .300 | .587 | .201 | 38 | .842 | .56000 | 2.79162 | -5.09134 | 6.21134 |
|  | Equal variances not assumed |  |  | .201 | 37.922 | .842 | .56000 | 2.79162 | -5.09173 | 6.21173 |

**Table 3. Outcomes obtained in subsequent speech tests (SRT values), with M and SD, in participants that started learning English earlier in life (<7) and later in life (>7).**

| **Test** | **start learning the language** | **N** | **Mean** | **Std. Deviation** | **Std. Error Mean** |
| --- | --- | --- | --- | --- | --- |
| A1 | Early | 17 | 20.0353 | 11.96160 | 2.90111 |
|  | Late | 17 | 26.0765 | 10.35287 | 2.51094 |
| ATm1 | Early | 17 | 13.8647 | 7.28740 | 1.76745 |
|  | Late | 17 | 19.2059 | 9.99090 | 2.42315 |
| ATnm1 | Early | 17 | 13.1824 | 10.37016 | 2.51513 |
|  | Late | 17 | 20.5471 | 8.41911 | 2.04193 |
| A2 | Early | 17 | 5.7765 | 7.31467 | 1.77407 |
|  | Late | 17 | 7.9118 | 7.20086 | 1.74646 |
| ATm2 | Early | 17 | .8882 | 6.50585 | 1.57790 |
|  | Late | 17 | 3.7118 | 6.26976 | 1.52064 |
| ATnm2 | Early | 17 | 8.8235 | 9.58218 | 2.32402 |
|  | Late | 17 | 11.4765 | 8.53306 | 2.06957 |

**Table 4.** **Outcomes obtained in subsequent speech tests (SRT values) in participants that started learning English earlier in life (<7) and later in life (>7) :** **the results of between-group comparisons (independent t-tests).**

|  | | Levene's Test for Equality of Variances | | t-test for Equality of Means | | | | | | |
| --- | --- | --- | --- | --- | --- | --- | --- | --- | --- | --- |
|  |  | F | Sig. | t | df | Sig. (2-tailed) | Mean Difference | Std. Error Difference | 95% Confidence Interval of the Difference | |
|  |  |  |  |  |  |  |  |  | Lower | Upper |
| A1 | Equal variances assumed | 2.942 | .096 | -1.575 | 32 | .125 | -6.04118 | 3.83683 | -13.85655 | 1.77420 |
|  | Equal variances not assumed |  |  | -1.575 | 31.355 | .125 | -6.04118 | 3.83683 | -13.86286 | 1.78051 |
| ATm1 | Equal variances assumed | 1.229 | .276 | -1.781 | 32 | .084 | -5.34118 | 2.99926 | -11.45046 | .76811 |
|  | Equal variances not assumed |  |  | -1.781 | 29.269 | .085 | -5.34118 | 2.99926 | -11.47290 | .79055 |
| ATnm1 | Equal variances assumed | 1.722 | .199 | -2.273 | 32 | .030 | -7.36471 | 3.23966 | -13.96368 | -.76574 |
|  | Equal variances not assumed |  |  | -2.273 | 30.704 | .030 | -7.36471 | 3.23966 | -13.97462 | -.75479 |
| A2 | Equal variances assumed | .141 | .710 | -.858 | 32 | .397 | -2.13529 | 2.48947 | -7.20618 | 2.93559 |
|  | Equal variances not assumed |  |  | -.858 | 31.992 | .397 | -2.13529 | 2.48947 | -7.20623 | 2.93564 |
| ATm2 | Equal variances assumed | .120 | .732 | -1.288 | 32 | .207 | -2.82353 | 2.19137 | -7.28721 | 1.64015 |
|  | Equal variances not assumed |  |  | -1.288 | 31.956 | .207 | -2.82353 | 2.19137 | -7.28745 | 1.64039 |
| ATnm2 | Equal variances assumed | .628 | .434 | -.853 | 32 | .400 | -2.65294 | 3.11194 | -8.99176 | 3.68588 |
|  | Equal variances not assumed |  |  | -.853 | 31.579 | .400 | -2.65294 | 3.11194 | -8.99508 | 3.68920 |

**Table 5. Outcomes obtained in subsequent speech tests (SRT values), with M and SD, in participants that rate their English command as higher (>4.17 on scale 1-5) and those that rate is as lower (≤ 4.17).**

| **Test** | **Self-rated English command** | **N** | **Mean** | **Std. Deviation** | **Std. Error Mean** |
| --- | --- | --- | --- | --- | --- |
| A1 | H | 20 | 27.0750 | 10.72105 | 2.39730 |
|  | L | 20 | 18.8350 | 9.84903 | 2.20231 |
| ATm1 | H | 20 | 19.0500 | 10.33958 | 2.31200 |
|  | L | 20 | 14.5750 | 7.38140 | 1.65053 |
| ATnm1 | H | 20 | 19.0600 | 7.90006 | 1.76651 |
|  | L | 20 | 14.2750 | 10.17809 | 2.27589 |
| A2 | H | 20 | 8.8750 | 6.74614 | 1.50848 |
|  | L | 20 | 4.0600 | 6.32858 | 1.41511 |
| ATm2 | H | 20 | 1.9050 | 6.50089 | 1.45364 |
|  | L | 20 | 2.2700 | 5.65137 | 1.26369 |
| ATnm2 | H | 20 | 10.7250 | 8.63340 | 1.93049 |
|  | L | 20 | 9.5950 | 8.99002 | 2.01023 |

**Table 6. Outcomes obtained in subsequent speech tests (SRT values) in participants that rate their English command as higher (>4.17 on scale 1-5) and those that rate is as lower (≤ 4.17): the results of between-group comparisons (independent t-tests).**

|  | | Levene's Test for Equality of Variances | | t-test for Equality of Means | | |
| --- | --- | --- | --- | --- | --- | --- |
|  |  | F | Sig. | t | df | Sig. (2-tailed) |
|  |  |  |  |  |  |  |
| A1 | Equal variances assumed | 0.086 | 0.771 | 2.531 | 38 | 0.016 |
|  | Equal variances not assumed |  |  | 2.531 | 37.730 | 0.016 |
| ATm1 | Equal variances assumed | 3.783 | 0.059 | 1.575 | 38 | 0.123 |
|  | Equal variances not assumed |  |  | 1.575 | 34.374 | 0.124 |
| ATnm1 | Equal variances assumed | 1.501 | 0.228 | 1.661 | 38 | 0.105 |
|  | Equal variances not assumed |  |  | 1.661 | 35.797 | 0.105 |

**Table 7. Outcomes of the Spearman’s Rho correlation analysis between outcomes in subsequent speech tests (SRT values) before training and use of the English language (in years).**

|  | A1 | ATm1 | ATmn1 |
| --- | --- | --- | --- |
| Correlation Coefficient | -0.292 | -0.264 | -0.362 |
| Significance (2-tailed) | 0.068 | 0.100 | 0.022 |
| N | 40 | 40 | 40 |
